# Digital health technologies for high-risk pregnancy management: three case studies using Digilego framework

**DOI:** 10.1093/jamiaopen/ooae022

**Published:** 2024-03-07

**Authors:** Sahiti Myneni, Alexandra Zingg, Tavleen Singh, Angela Ross, Amy Franklin, Deevakar Rogith, Jerrie Refuerzo

**Affiliations:** Department of Clinical and Health Informatics at McWilliams School of Biomedical Informatics, The University of Texas Health Science Center at Houston, Houston, TX 77030, United States; Department of Clinical and Health Informatics at McWilliams School of Biomedical Informatics, The University of Texas Health Science Center at Houston, Houston, TX 77030, United States; Department of Clinical and Health Informatics at McWilliams School of Biomedical Informatics, The University of Texas Health Science Center at Houston, Houston, TX 77030, United States; Department of Clinical and Health Informatics at McWilliams School of Biomedical Informatics, The University of Texas Health Science Center at Houston, Houston, TX 77030, United States; Department of Clinical and Health Informatics at McWilliams School of Biomedical Informatics, The University of Texas Health Science Center at Houston, Houston, TX 77030, United States; Department of Clinical and Health Informatics at McWilliams School of Biomedical Informatics, The University of Texas Health Science Center at Houston, Houston, TX 77030, United States; Department of Obstetrics, Gynecology, and Reproductive Sciences at McGovern Medical School, The University of Texas Health Science Center at Houston, Houston, TX 77030, United States

**Keywords:** women’s health, high-risk pregnancy, digital health, social media

## Abstract

**Objective:**

High-risk pregnancy (HRP) conditions such as gestational diabetes mellitus (GDM), hypertension (HTN), and peripartum depression (PPD) affect maternal and neonatal health. Patient engagement is critical for effective HRP management (HRPM). While digital technologies and analytics hold promise, emerging research indicates limited and suboptimal support offered by the highly prevalent pregnancy digital solutions within the commercial marketplace. In this article, we describe our efforts to develop a portfolio of digital products leveraging advances in social computing, data science, and digital health.

**Methods:**

We describe three studies that leverage core methods from *Digilego* digital health development framework to (1) conduct large-scale social media analysis (*n* = 55 301 posts) to understand population-level patterns in women’s needs, (2) architect a digital repository to enable women curate HRP related information, and (3) develop a digital platform to support PPD prevention. We applied a combination of qualitative coding, machine learning, theory-mapping, and programmatic implementation of theory-linked digital features. Further, we conducted preliminary testing of the resulting products for acceptance with sample of pregnant women for GDM/HTN information management (*n* = 10) and PPD prevention (*n* = 30).

**Results:**

Scalable social computing models using deep learning classifiers with reasonable accuracy have allowed us to capture and examine psychosociobehavioral drivers associated with HRPM. Our work resulted in two digital health solutions, MyPregnancyChart and MomMind are developed. Initial evaluation of both tools indicates positive acceptance from potential end users. Further evaluation with MomMind revealed statistically significant improvements (*P* < .05) in PPD recognition and knowledge on how to seek PPD information.

**Discussion:**

Digilego framework provides an integrative methodological lens to gain micro-macro perspective on women’s needs, theory integration, engagement optimization, as well as subsequent feature and content engineering, which can be organized into core and specialized digital pathways for women engagement in disease management.

**Conclusion:**

Future works should focus on implementation and testing of digital solutions that facilitate women to capture, aggregate, preserve, and utilize, otherwise siloed, prenatal information artifacts for enhanced self-management of their high-risk conditions, ultimately leading to improved health outcomes.

## Introduction

Maternal health is of significant importance for individuals, families, and societies as a whole. Maternal health refers to the health of women during pregnancy, childbirth, and the postpartum period, as well as the health of the newborn.^1–4^ The United Nations has included maternal health as one of the sustainable development goals, which aims to enhance investments that address maternal mortality, access to reproductive health services, and overall women’s health disparities.^2^ In line with this effort, the National Institutes of Health has launched their program, Implementing a Maternal health and PRegnancy Outcomes Vision for Everyone (IMPROVE) to reduce preventable causes of maternal deaths and improve health for women before, during, and after delivery with special emphasis on health disparities.^5^

American women have the greatest risk of dying from high-risk pregnancy (HRP) complications among 11 high-income countries.^5^ Gestational diabetes mellitus (GDM), hypertension (HTN), and peripartum depression (PPD) are some of the most common medical disorders of HRPs. It is estimated that 8%-14% of pregnancies (depending on diagnostic criteria) are complicated with any type of diabetes and that approximately 86% of these cases are GDM.^6^^,^^7^ GDM affects more than 20 million live births, or approximately one in six births, worldwide (8-10). Pregnancy complications of GDM include preeclampsia (9.8%-18%), development of overt type 2 diabetes mellitus (70%) and cesarean section (17%-25%). Other complications include vascular dysfunction, non-alcoholic fatty liver disease, dyslipidemia, chronic kidney disease, and ischemic heart disease.^8^^,^^9^ When a pregnancy is associated with new- onset HTN, this can lead to preeclampsia, which occurs after 20 weeks of pregnancy and frequently near term. In the United States, preeclampsia has seen a 7-fold increase over the past two decades.^10^ Pregnancy complications of HTN in pregnancy include fetal growth restriction, oligohydramnios, placental abruption, non-reassuring fetal status, and spontaneous or medically indicated preterm delivery. HTN contribute to 16% of all maternal deaths.^10^^,^^11^ Similarly, PPD is a serious public health problem that affects a significantly high number (approximately 10%-15%) of peripartum women.^12^ PPD is associated with pregnancy complications such as low birth weight and preterm births.^12^^,^^13^ Women with PPD may also forego recommended prenatal checkups, immunization schedules for the baby, and well-baby check-ups.^14^ If left untreated, PPD can result in longer and more intense episodes of depression for the mother, and later on with cognitive and behavioral issues for the baby.^15^^,^^16^ In addition to the health risks, GDM, HTN, and PPD are among the leading cost drivers in adult women based on the highest cost reported, thereby increasing the overall economic burden of pregnancy on the US health care system.^17–19^

**Figure 1.**
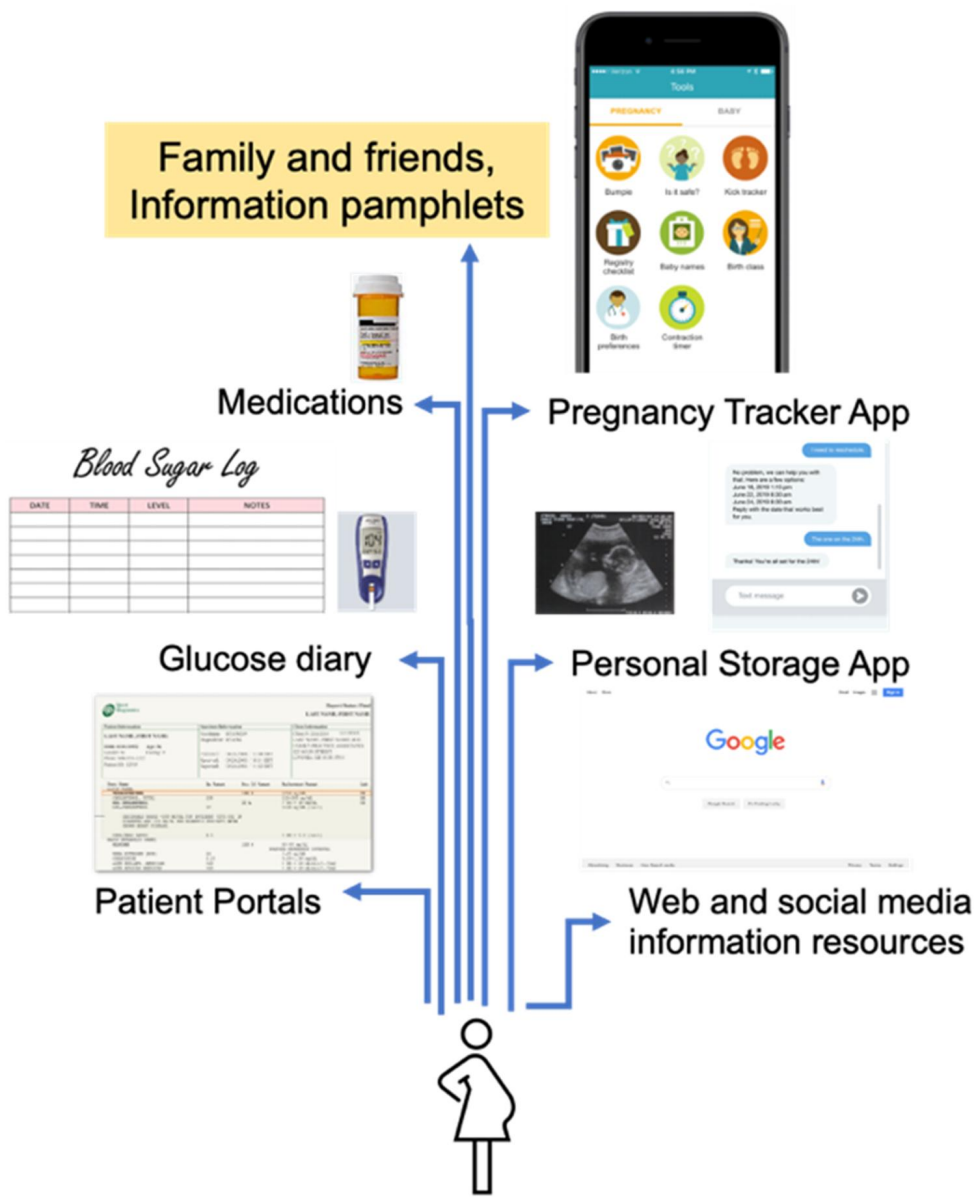
Patient-facing information resources for high-risk pregnancies with gestation diabetes mellitus.

Outcomes of these HRPs are heavily dependent on active patients’ engagement in their pregnancy. Along with close fetal surveillance, maternal behavior modification is often seen as an effective strategy for HRP management (HRPM).^20^^,^^21^ For instance, for GDM, modified dietary interventions, exercise, glucose monitoring, and/or medications result in improved outcomes in maternal glycemic control and birth outcomes.^22–24^ As shown in Figure 1, oftentimes, critical information needed for patient engagement is distributed in the form of prenatal ultrasounds, discharge summary, lab reports in paper and electronic formats across personal health records, text messages, and individual websites (eg, educational resources). Such scattered and incoherent HRP information overload coupled with the steep learning curve associated with sustaining behavior modifications, and conflicting information resources (eg, physician recommendations, social media influencers) lead to response fatigue, limiting their self-efficacy and engagement in GDM.^25^^,^^26^ Recent trends highlight the utility of digital health as a promising solution to the increasing prevalence and complexity in engaging patients in health behaviors across the spectrum (eg, medication adherence, nutrition).^27–32^

While the current digital market place for women is crowded,^33–36^ there are hardly any solutions focused on integrative HRPM, let alone digitally inclusive disparities-mending personalized knowledge ecosystems.^37^ Little is known about mechanisms through which multilevel inter- and intra-personal factors, individualized knowledge resources, and theory-driven behavior change techniques can be packaged into comprehensible, engaging, and effective digital health solutions for enhancing maternal health.^28^^,^^38^ New approaches are needed that model the dynamics of the social context (eg, family ties, social influence) and individual factors (eg, health beliefs, cognitive heuristics) affecting critical decision making and opinion formation, to develop interventions that drive patient activation and engagement in effective utilization of digital platforms for HRPM.^29^ In addition, few digital health development frameworks facilitate cross-disciplinary integration beyond computational programming and oftentimes competing interests involved in design choices and feature trade-offs affects community-engaged technology development efforts.^30^^,^^31^ This lack of consideration and accommodation poses a risk for not only the failure of these technologies, but their failure to address women’s needs, importantly, especially the specialized needs of populations plagued with health disparities (eg, Hispanic women who are at the highest risk for GDM).^39^^,^^40^ To fill these gaps, in this article, we present and apply Digilego, a novel digital health development framework,^41^^,^^42^ which integrates advances in digital health and data science through a combination of theory-guided qualitative research, advances in social computing, and patient engagement models.

## Methods

### *Digilego* framework

**Figure 2.**
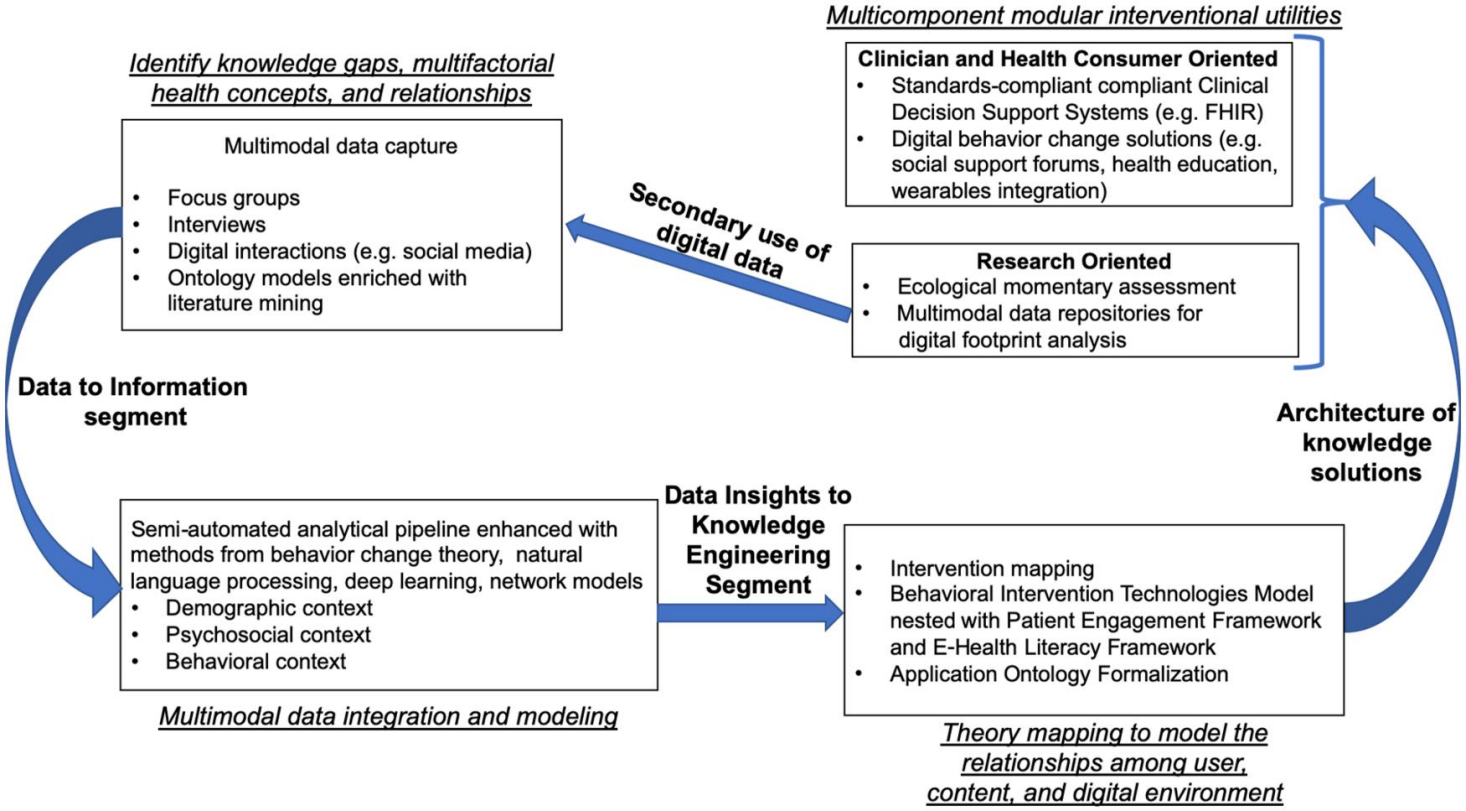
High-level overview of Digilego modular architecture and design pipeline.

The overview of our generalized methodological framework, Digilego, is illustrated in Figure 2. Digilego facilitates the development and integration of individual *Digilego* building blocks to form a patient-oriented digital health solution.^41^^,^^42^ The unique features of these Digilego blocks are their reusability and customization abilities according to care context. These Digilego blocks can be put together to build digital health solutions that are modular, flexible and extensible, yet comprehensive. Such compartmentalized design architecture of the Digilego framework allowed us to facilitate customization of health education, harness machine-based querying, integrate digital engagement tactics, and adopt theory-driven techniques that ultimately enable the development of women-empowering flexible digital health solutions that can be used and modified across the care continuum for efficient care coordination and self-health management. Our Digilego framework is *theory-guided:* drawing links between behavior change theories, naturalistic decision making models, and multilevel inter- and intrapersonal influences on HRPM; *empirically informed*: needs gathering based on large-scale population level social listening among diverse populations as well as qualitatively enriched contextual inquiry using interviews with pregnant women from different ethnic groups and diverse income groups, thereby encompassing digital footprints and real-world evidence, *inclusive:* community engagement through interviews to obtain perspectives of women to understand individual-level, interpersonal, and health system-level factors affecting their engagement in perinatal health management, *translational*: providing guided learning artifacts responsive to patient care contexts in digital settings.

We have instantiated Digilego to develop a portfolio of digital health technologies to enable patient engagement in prevention and management of high-risk conditions during peripartum period in a series of studies described in this article. In Study 1, we focused on gathering population needs for PPD prevention and management, in Study 2, we architected a specialized personal health library for management of GDM and HTN, and in Study 3 we developed and tested a state-of-the art digital tool for prevention of PPD. We adopted a mixed methods approach across the three studies utilizing multiple methodological components outlined in Figure 2. These methods include, focus groups, semi-structured interviews, semi-automated methods of online peer interactions, theory mapping using Behavioral Interventions Technology (BIT) model^43^ and optimizing digital experiences using Patient Engagement Framework (PEF)^44^ which we describe below in detail. In order to enrich our understanding of women’s needs, we took a phased approach, starting with large-scale social media analysis at population-level interweaved with focus groups and semi-structured interviews to capture inter- and intra-personal factors affecting women’s engagement in HRPM through digital resources at individual-level. The focus groups allowed us to understand shared concerns, while interviews allowed us to capture patient journey and needs, and online peer interactions allowed us to explore generalizability of our design solutions and also examine solution seeking behaviors of women with HRPs, which can in turn help us in theory-mapping and content and feature engineering as well as engagement sustenance as we design digital pathways for HRPM.

#### Semi-automated methodology to enable large-scale social listening

Data extraction: For Study 1, we analyzed peer interactions extracted from two popular pregnancy apps used by women on a world-wide scale, What to Expect^45^ and Baby Center.^46^ Peer interactions were extracted from the PPD-specific forums “Postpartum Depression” (What to Expect) and “Postpartum Depression, Anxiety, and Related Topics” (Baby Center). We used a web scraping software called Scrapy^47^ for extracting the messages from the above-mentioned forums. We obtained a total of 12 416 threads containing 55 301 messages exchanged by 9364 individuals from years 2008 to 2022. The social forums and their respective messages used in this study were marked public and we also removed any potential identifiers (ie, usernames) from our data set. This study was approved by the University of Texas Health Science Center’s Committee for the Protection of Human Subjects IRB number HSC-SBMI-15-0697.

Qualitative analysis: From the above data set, a total of 1424 posts were randomly selected for qualitative analysis to assign thematic labels that were derived from grounded theory approaches^48^ (see Table S1 for definitions of themes and sample messages). These labels were guided by open codes that emerged from the data (eg, baby, panic, medication), from which we first developed a total of 14 themes, (eg, physical pain, anxiety and stress, baby routine, breastfeeding). These themes were then merged axially to form six themes (eg, physical and mental, mother and infant dyad) to facilitate pragmatic interventional insights in terms of content and feature optimization. In order to ensure consistency in the labeling process, the messages were labeled by two coders using a subset of 150 messages and computing interrater reliability among them (Cohen’s Kappa). Interrater reliability was consistently substantial across all themes, with the highest Cohen’s Kappa measure of 1.00 in the theme of “Family and friends,” followed by “Mother and Infant Dyad” (*k* = 0.98), “Doctor and Patient Dyad” (*k* = 0.92), “Physical and Mental Health” (*k* = 0.85), “Medications” (*k* = 0.84), and “Social Support” (*k* = 0.84). Any disagreements regarding labels were discussed among the researchers until a mutual agreement was reached.

**Figure 3.**
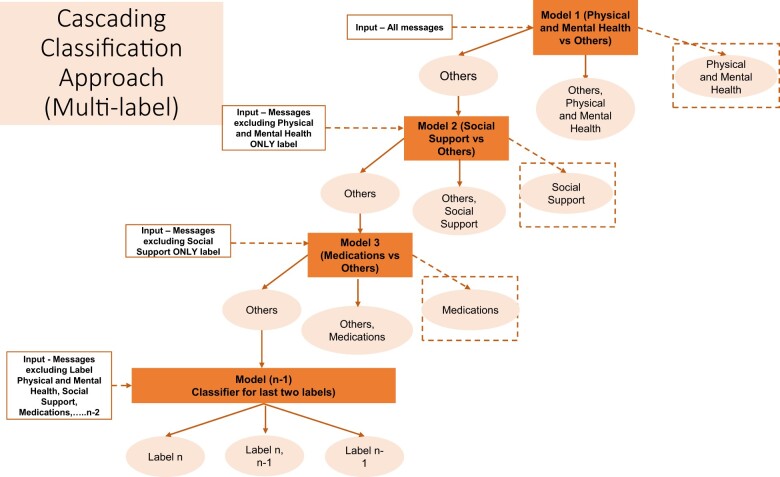
Figure 3 shows the cascading algorithm employed for multilabel classification of messages exchanged in an online forum on different topics ranging from physical health, social support to medications.

Automated text analysis: Transformer-based language models have emerged as the state-of-the-art models in many Natural Language Processing (NLP) tasks primarily because of their ability to capture bidirectional contextual information.^49^^,^^50^ Bidirectional Encoder Representations from Transformers (BERT) is a multilayer transformer encoder model implemented using the self-attention mechanism that learns information from both left and right side of token’s context.^49^^,^^50^ For this study, we further pretrained BERT (called BERT PPD-trained) in order to perform theme classification of messages from PPD online health communities. We pretrained the BERT-base model on PPD-specific corpus using our unlabeled data set of 55 301 messages. Given the imbalanced distribution of thematic labels in our data set, we built sequential models using the cascading classification approach based on the prevalence of labels in the manually annotated data set (Figure 3). We used a fine-tuning layer that consisted of two fully connected dense layers (768 and 512 units, respectively) and a sigmoid activation function in the output layer (2 units). We set the maximum sequence length to 100 and batch size to 32. We used AdamW optimizer to find individual learning rates for each parameter with the learning rate of 1 × 10^−5^. We then split the entire data set into 80%, 10%, and 10% for training, validation, and test sets, respectively. The model was trained for 20 epochs to fine-tune for our classification task. We used three evaluation metrics (recall, precision, and F1-score) to evaluate the classifier’s predictions per class on the held-out test data set.

#### Development and optimization of digital experiences for HRPM

Following the Digilego framework, for Studies 2 and 3, we used a combination of focus groups and interviews to gather women’s needs for HRPM in addition to aforementioned social listening to understand population-level perspectives. Details of the methods and subsequent results can be found in our published works.^51^^,^^52^ In order for us to achieve theoretically informed digital health products for HRPM, we utilized BIT model.^43^ This allowed us to operationalize behavioral theory into tangible digital features. This model prompts intervention developers to answer the questions of Why?, How? (conceptual and technical), What?, and When? at the time of technology development. Further, to facilitate the identification of specific behaviorally based techniques to be integrated into digital modalities, we leveraged Michie’s Behavior Change Taxonomy.^53^ This is a standardized, comprehensive taxonomy of 93 theory-linked techniques for use in health behavior change interventions. Utilizing PEF, we defined the intended digital engagement level and optimized the user interaction features and technical complexity on the backend of the solutions allowing us to make informed choices and tradeoff decisions among theoretical nuances, technological sophistication and user experience producing a modular digital roadmap for our intended use cases in HRPM. To create digital features specific to GDM, HTN, and PPD, we leveraged and repurposed existing Digilego core architecture,^41^^,^^42^^,^^54^ specifically DigiSocial and DigiConnect blocks to provide coverage for peer support, journaling, and patient-generated data. Multiple specialized Digilego blocks were conceived and implemented (eg, DigiCAPSULE, DigiNavi). These special purpose features enable women to capture, aggregate, preserve, and summarize their HRP information and were dedicated for onboarding and streamlining digital experiences for women during the perinatal period. This mapping process allowed us to produce two digital products for HRPM, MomMind (for PPD) and MyPregnancyChart (for GDM/HTN). Initial prototypes for these applications were developed using AdobeXD and proof-of-concepts were built using ReactJS, Django, RedCap, and MySQL.

#### Initial evaluation of patient acceptance of MyPregnancyChart and MomMind

Our evaluation consisted of is combination of surveys and individual interviews with perinatal women (*n* = 10 for MyPregnancyChart) in Study 2 and (*n* = 30 for MomMind) in Study 3. Individuals had to be at least 18-years-old, English-speaking, and currently seeking pre/post-natal care at The Fetal Center clinic located within the UT Physicians healthcare system in the Texas Medical Center (Houston, Texas) to be eligible for participation. Eligible individuals were approached by their OB/GYN provider and explained the study. Informed consent was obtained for those who agreed to participate in the study. A $25 gift card for use in a national-level department store chain was offered as incentive for participating in the evaluation. The studies were approved by the Institutional Review Board of the Center for Protection of Human Subjects at the University of Health Science Center at Houston, HSC-SBMI-22-0750 and HSC-SBMI-16-0594. Individuals were asked to complete a survey on their demographic background, their information technology preferences, and their prior experiences with HRPs. A semi-structured interview was conducted with questions based on the Integrated Behavior Model,^55^ a model that focuses on individual’s perceptions and intentions regarding a specific health behavior. Acceptability of the application was measured using Weiner’s set of Likert-scale surveys for intervention acceptance, feasibility, and appropriateness measures.^56^ In addition to these, for MomMind evaluation, we conducted an assessment of individuals’ PPD health literacy (knowledge, attitudes, beliefs) was conducted pre-and-immediately-post use of the app using the Postpartum Depression Literacy Scale.^57^ Also, we evaluated the education content presented through the “PPD 101” videos using a questionnaire based on previous evaluations of educational content presented in a similar digital format.^58^ The qualitative and quantitative methods used during our evaluation are summarized in Table S1.

## Results

The findings from our Study 1 are as follows. The theme of “Physical and Mental Health” was the most discussed among users of PPD online social forums, with a total of 547 unique users. This was followed by the theme of “Social Support” (*n* = 437), “Medications” (*n* = 423), “Mother and Infant Dyad” (*n* = 359), “Doctor and Patient Dyad” (*n* = 335), and “Family and Friends” (*n* = 322). Detailed definitions and examples of peer interactions can be found in Table S2. To illustrate how these themes are applied in the context of the social forum, we follow a single conversation thread in this example. User 1 has begun the thread by expressing her frustration at the significant anxiety symptoms she experienced soon after starting a medication:

*I started taking Zoloft a wk and half ago and my anxiety has been through the roof. I haven’t been able to drive since I started taking it out of fear of having a panic attack…I know you have to give it 2 wks but I can’t keep having extreme panic attacks every day. Help I don’t know what to do.*

The user has sought peer support for help in deciding whether to stop the medication immediately, or continue for the recommended time of 2 weeks before stopping. The original post contains the themes of “Medications” and “Physical and Mental Health.” The replies to the original post contain a variety of themes, from “Social Support” in encouraging User 1 to continue the medication for 2 weeks, to “Doctor and Patient Dyad” in recommending the user seek care from doctors specializing in peripartum depression. An example reply illustrating social support:

*Please give it time to work! I am on lexapro and I had a similar experience. I needed the 2 weeks before I started feeling a million times better. My only complaint now is the emotional numbness you’re referencing.*

In analyzing this conversation thread, we can see how users’ interactions and the themes they discuss in the forum can help shape their decisions regarding PPD management. As shown in Table 1, the “Medications” theme had the highest F1 score of 0.96, followed by “Mother and Infant Dyad” theme which had a F1 score of 0.93, and “Family and Friends” theme had an F1 score of 0.90. The performance for “Doctor and Patient Dyad” was also substantial with an F1 score of 0.89, as was the performance for “Physical and Mental Health” with an F1 score of 0.88. The performance for “Social Support” was the lowest with an F1 score of 0.75.

**Table 1.** Overall model performance of domain-trained BERT classification.

BERT (PPD-trained)			
Overall model performance			
Class	Precision	Recall	F1
Physical and Mental Health	0.84	0.92	0.88
Social Support	0.73	0.78	0.75
Medications	0.94	0.99	0.96
Mother and Infant Dyad	0.89	0.99	0.93
Family and Friends	0.93	0.88	0.90
Doctor and Patient Dyad	0.87	0.91	0.89

The results of our development efforts for MyPregnancy Chart and MomMind can be found in Figure S1 and Table S3. Our initial evaluation of our MyPregnancyChart prototypes in Study 2 resulted in firm acceptance from our participants as indicated in Figure S2. Nine out of 10 participants indicated the solution is practical and approved the use of images-driven user interface for library interactions, and expressed the need for advanced annotation features rather than manual completion of curated information artifacts for GDM/HTN such as photos of blood glucose and blood pressure logs. All participants agreed the interface navigation is straightforward and intutive. Initial evaluation of MomMind in Study 3 revealed 96.6% of the participants approved of MomMind (29 out of 30), 96.6% of the participants deemed MomMind is easy to use (29 out of 30), 93.3% deemed MomMind is a good match (28 out of 30), and the PPD 101 content was deemed acceptable by 90% of the participants (27 out of 30). Our results also revealed statistically significant results in participants’ ability to recognize PPD pre- (3.89/5) and post- (4.25/5) MomMind use. Similar improvements are noted in their knowledge to seek PPD information (pre (3.49/5), post (3.79/5)).

## Discussion

Our study provides an introduction to cross-cutting methods from behavioral theory, social sciences, data science, and digital health development, and describes their application to identify and address women’s needs for HRPM through a set of three studies. With the onset of mobility and connectivity in the communication sector, peer interactions in online health communities reflect the intricacies of engagement in HRPM as experienced in real-time at individual, community, and societal levels.^59–62^ Existing theories of behavior change and patient engagement models suggest a myriad of content-driven strategies to elicit specific socio-behavioral mechanisms beyond social support (eg, stimulus control, observational learning, role models) to drive maternal behavior modification and engagement.^53^^,^^63^ Through our Study 1, we demonstrate the utility of peer interactions in online communities to understand women’s needs from established participatory venues in the digital domain. These results along with our focus groups and user interviews with women dealing with HRPs^51^^,^^52^ have facilitated our development efforts resulting in two digital applications—MomMind and MyPregnancyChart focusing on various aspects of maternal health management. Insights extracted from social media analysis have helped us with content framing, design optimization, and feature implementation for self-monitoring of health behaviors in both MomMind and MyPregnancyChart.^64–66^ MomMind^54^^,^^64^ focuses on journaling and health education for women seeking perinatal care, and MyPregnancyChart focusses on user generated image repository of cataloguing perinatal health information as they manage GDM/HTN. Peer interactions illuminated specific knowledge gaps, such as misconceptions surrounding infant formula use, a significant source of anxiety for new mothers with PPD, which we directly addressed through tailored content creation within our MomMind application. In contrast, the unique needs of women with GDM/HTN prompted the integration of a health education module within our MyPregnancyChart app, specifically to debunk prevalent myths and resolve prevalent uncertainties regarding health supplements (eg, alternatives to insulin therapy). While discussions on PPD are more geared towards emotional needs and societal expectations, GDM/HTN discussions are focused on behavior modification, reasoning for physical symptom manifestations (eg, systolic spikes, adamant fasting glucose), and relevance of physician-prescribed behavioral goals. Our results from preliminary evaluation of MyPregnancyChart in Study 2 have revealed women’s acceptance of image-based health library, and emphasized the need for automated methods for machine-based annotation, integrative summaries, and personalized health education taking into account clinical, social, and demographic contexts of women, while accounting for health literacy and behavioral support to self-monitor maternal and neonatal health. Our evaluation results for MomMind in Study 3 suggest positive acceptance by women, including minority and low-income populations represented in our sample. During interviews, individuals identified many benefits of our applications, including creditable health information, ability to socialize with others, and recognized the synchronous design details with other general domain application they use in daily lives. Individuals also deemed the applications present simple design and ease of navigation, both seen as positive characteristics. However, participants noted room for improvement including interactive educational components with conversational capabilities, automated annotation capabilities for patient-generated data and images, and opportunities for practice-based peer support groups, which concurs with existing studies.^67^^,^^68^ Our paper has several limitations. In Study 1, the communication themes are limited to two online forums. And, the manual codes were inductively derived from only a subset of the data, additional themes might be present in the remainder of the data set. In Study 2, our evaluation is limited to preliminary prototypes with a low patient sample (*n* = 10) in formative phases and initial user acceptance of the proposed library solution for HRPM. Future studies testing the effectiveness of the solution for psychosocial measures (eg, self-efficacy, stress), knowledge level (eg, health literacy), pregnancy outcomes (eg, cesarean rates), and fetal outcomes (eg, birthweight) are highly desirable. In Study 3, participants had a limited time of interaction with MomMind, and a longitudinal study and log file analysis may provide a more thorough assessment of participant’s experiences. Further, a longitudinal study evaluating the efficacy and effectiveness of MomMind on PPD prevention and management is warranted.

## Conclusions

Maternal health management forms a crucial component of our societal wellness going well beyond the peripartum period affecting women, their families, and future generations.^69^ Better efficiency and effectiveness of digital solutions that enable prevention and management of high-risk pregnancies are vital to enhance the quality of life during and after pregnancy as well as improve pregnancy and fetal health outcomes.^70^ Our Digilego framework provides a foundational step that will help influence the development of digital applications with reusable and customizable components as per the needs of the women across the care continuum. While our framework is aimed at women’s specific health conditions, our methods and core infrastructure can be used to develop interactive and innovative digital care pathways for management of any chronic disease (eg, diabetes, cardiovascular health) that requires patient engagement and self-health management. Use of informatics-driven methods, behavior change theories, and patient-centered design approaches may help us develop scalable and cost-effective digital assets and products that can be deployed globally.

## Supplementary Material

ooae022_Supplementary_Data

## Data Availability

Some of the underlying data for this article are available upon request given participant privacy and data security needs.
