# Supplementary material for: Digital health technologies for high-risk pregnancy management: three case studies using Digilego framework
Source: JAMIA Open. 2024 Mar 7;7(1):ooae022. doi: 10.1093/jamiaopen/ooae022 (PMC10919928; doi:10.1093/jamiaopen/ooae022)
Supplement: ooae022_Supplementary_Data [file ooae022_supplementary_data.docx]

**Supplementary Materials**

Table 1- Instruments used during “MomMind” evaluation

| **Quantitative Instrument** | Main Outcome |
| --- | --- |
| Postpartum Depression Literacy Scale (3) | PPD Health Literacy |
| Weiner’s Intervention Acceptance, Feasibility, and Appropriateness Measure (5) | Population acceptance of MomMind. |
| “PPD 101” Questionnaire (4) | Feedback on MomMind education content. |
| **Qualitative Instrument** |  |
| Semi-structured interview (2) | Personal opinions and feedback regarding MomMind. |

Table 2. Examples of PPD thematic labels from social media

| Category | Definition | Sample Post |
| --- | --- | --- |
| Family and Friends | Posts where user specifically mentions family and/or close friends as an important factor in their mental health state. Role of family and friends can be direct or undirect, and positive or negative. | *“I started by just admitting it to my sisters whom I'm close with and then my husband. I surprisingly got a lot of love and encouragement and support…”.* |
| Medications | User statements or queries about pharmacological treatments for peripartum depression. | *“I'm 34 weeks and my Psych doctor recently prescribed Buspar 5 mg for myself…just wondering if anyone else has taken this Med during pregnancy and what their experience with it was ??”* |
| Physical and Mental Health | User describes changes in their body or state of mind during the peripartum period | *“I have anxious spells- with severe symptoms where I feel like I'm in a dream, panicky, heart racing, thoughts that I know I don't actually think. This can go on all day.”* |
| Mother and Infant Dyad | Posts where user describes their interactions and relationship with their infant, of both positive and negative nature | *“When I stopped breastfeeding it was like all this pressure went away and I felt more relaxed and I could concentrate on getting better.”* |
| Social Support | Content where a user provides support to their peers, and can be of four types: emotional (words of encouragement and kindness), appraisal (feedback on a situation), instrumental (a practical tool), and informational (personal experiences or educational resources). | *“[…] believe me you are not alone. I think you should get a second opinion honestly. I wish you the best of luck. Maybe try counseling too. Hang in there, you will be great!!!”* |
| Doctor and Patient Dyad | User interactions with their care providers as part of their mental health management | *"I'm only 16 days pp and after discussing the severity of my baby blues with my Obgyn yesterday, he prescribed Zoloft. My question is: is it too soon to assume ppd?[…]"* |


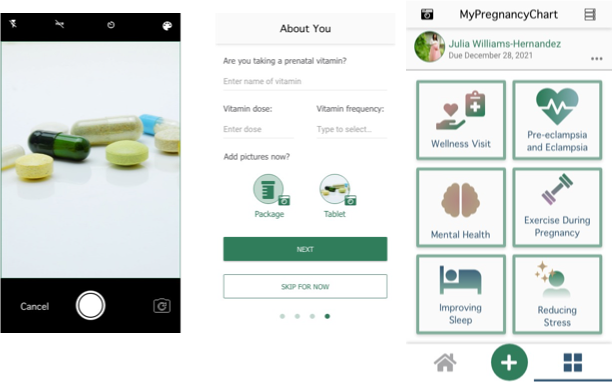


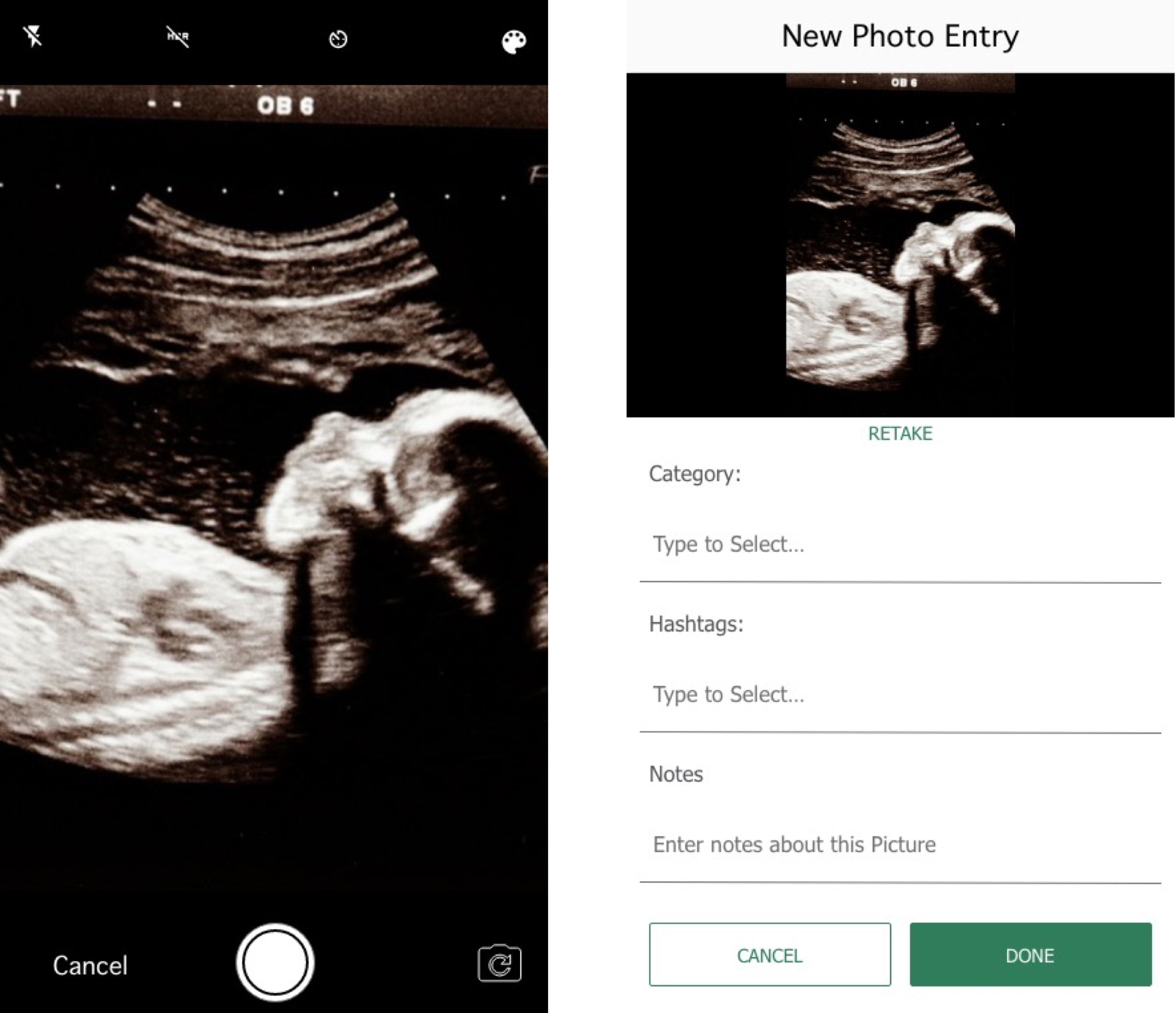


Figure 1. MyPregnancyChart Prototypes

Table 3- MomMind Features and content

| 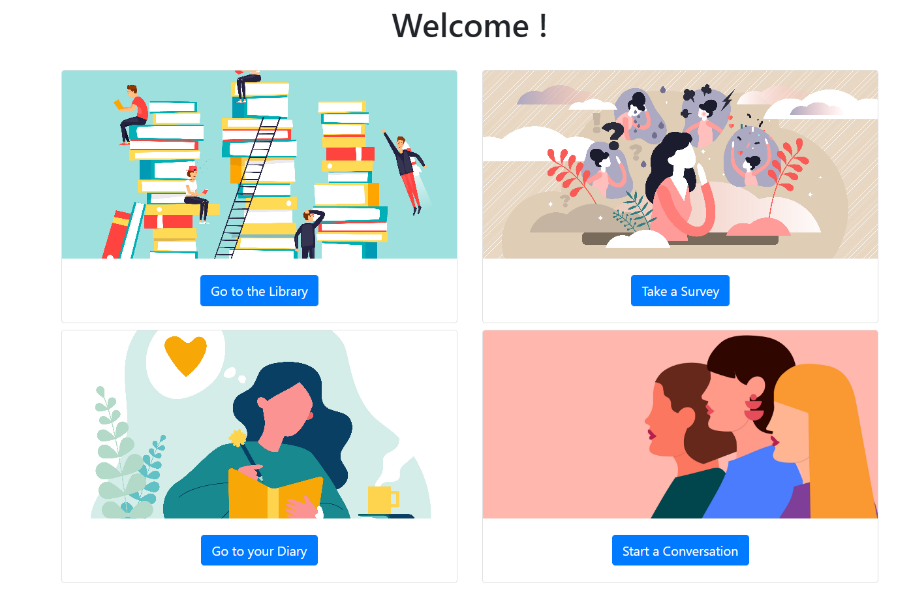 | MomMind homepage with access to the four modules: My Library, My Surveys, My Diary, and MomTalk. |
| --- | --- |
| 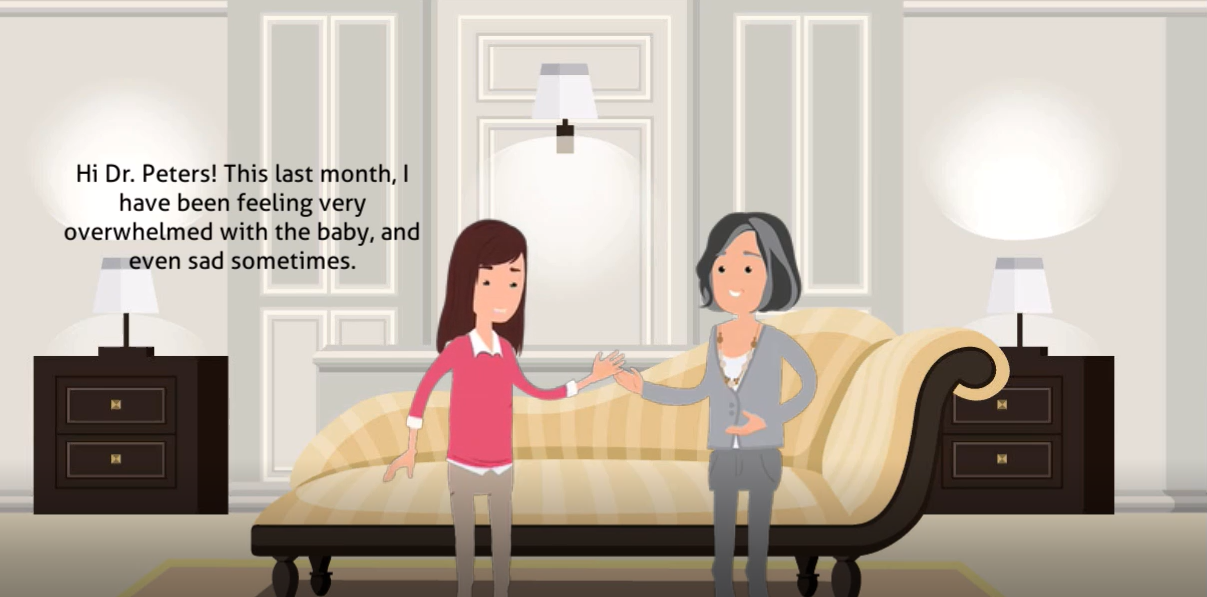 | “PPD 101” video implementing the behavior change technique of shaping knowledge through information about antecedents. |
| 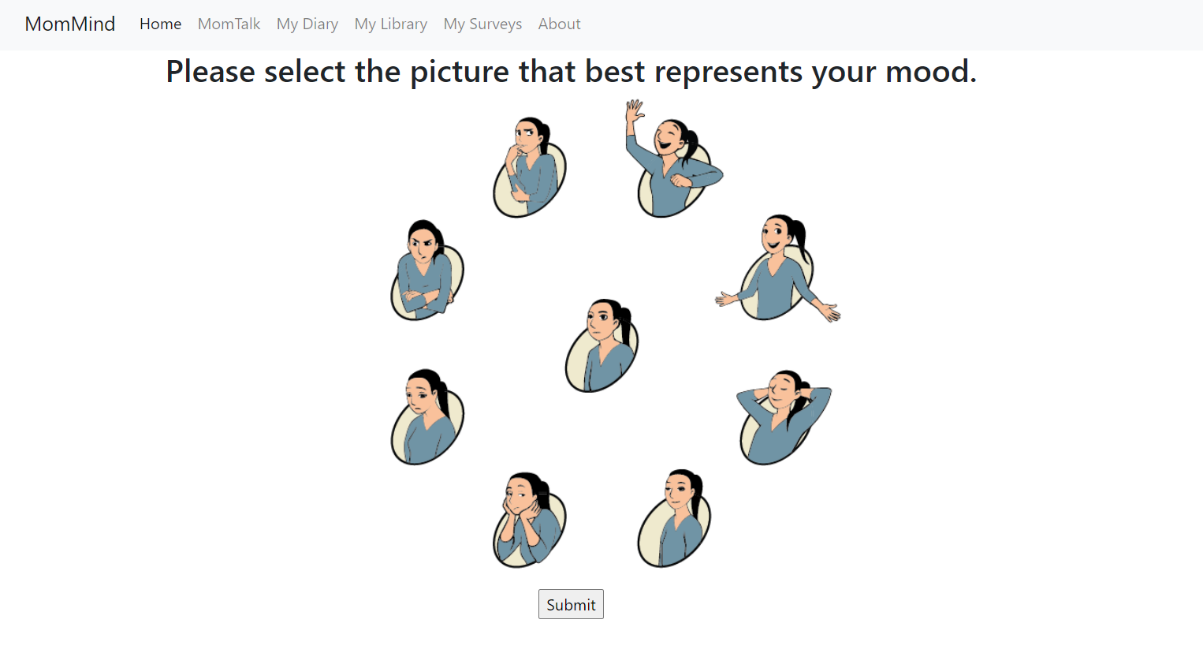 | Example of the Pick-a-Mood mood tracking survey. |


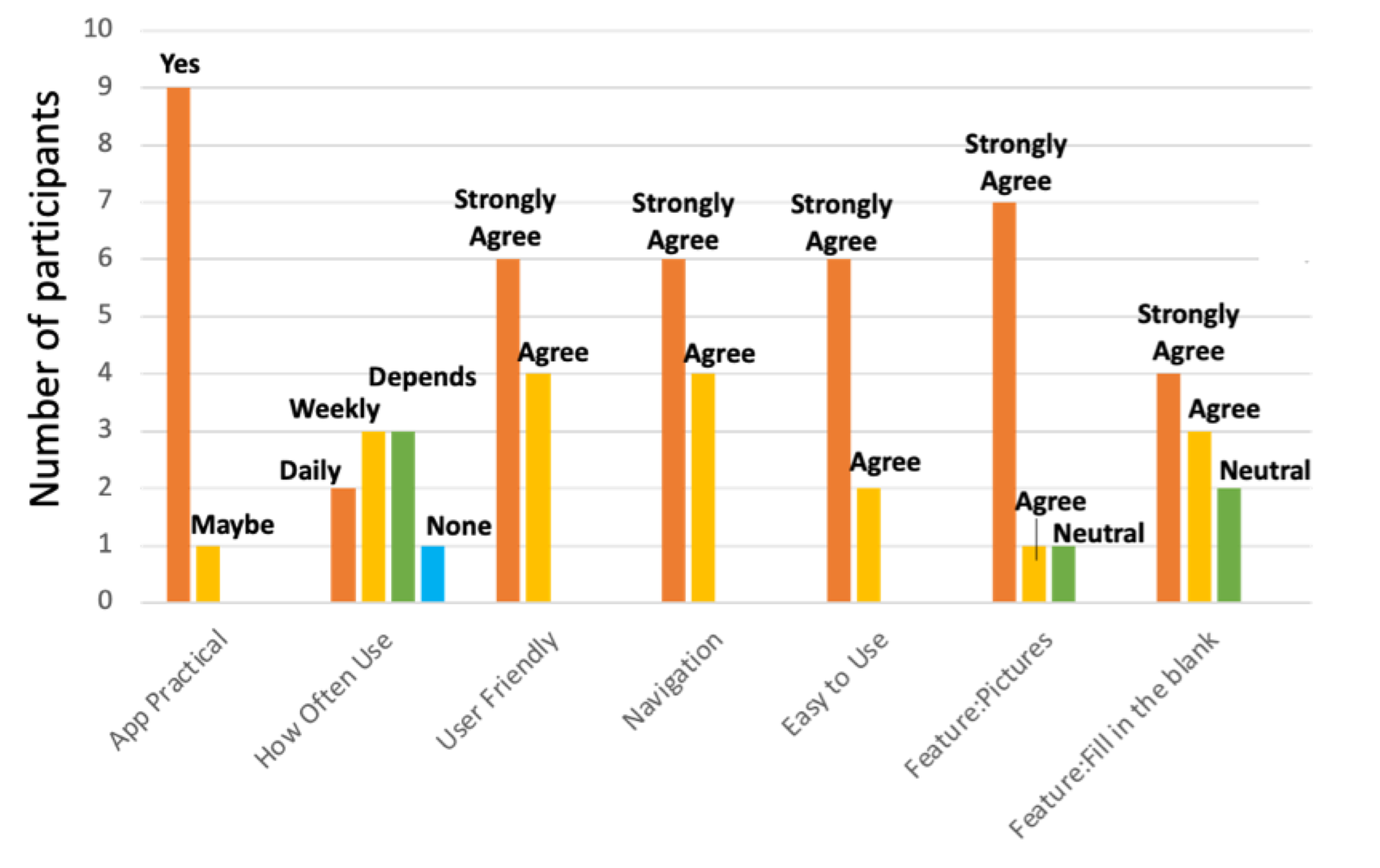


Figure 4. Initial acceptance testing for MyPregnancyChart.
